# Supplementary material for: Post-Diagnosis Dietary Patterns among Cancer Survivors in Relation to All-Cause Mortality and Cancer-Specific Mortality: A Systematic Review and Meta-Analysis of Cohort Studies
Source: Nutrients. 2023 Sep 4;15(17):3860. doi: 10.3390/nu15173860 (PMC10490392; doi:10.3390/nu15173860)

Title: Post-diagnosis dietary patterns among cancer survivors in relation to all-cause and cancer-specific mortality: a systematic review and meta-analysis of cohort studies

Authors: Maria-Eleni Spei [1], Ioannis Bellos [1], Evangelia Samoli [1], Vassiliki Benetou [1]

Supplementary Material

Contents

Supplementary S1: Search strategy 3

Supplementary S2: Excluded studies 4

Supplementary S3: A priori dietary patterns and all-cause mortality 7

SF S1: Galbraith's plot........78

SF S2: Funnel plot of a priori dietary patterns and all-cause mortality 87

Supplementary S4: A priori dietary patterns and cancer-specific mortality 9

SF S3: Forest plot of a priori dietary patterns and cancer-specific mortality by cancer type and overall........9

SF S4: Funnel plot of a priori dietary patterns and cancer-specific mortality among all studies….10

Supplementary S5: A priori dietary patterns and cancer-specific mortality - Further analysis of breast cancer studies 11

F S5: Funnel plot of a priori dietary patterns and breast cancer mortality 11

Supplementary S6: A priori dietary patterns and cancer-specific mortality - Further analysis of colorectal cancer studies 12

SF S6: Funnel plot of a-priori dietary patterns and colorectal cancer mortality 12

Supplementary S7: A-posteriori dietary patterns and cancer-specific mortality 13

SF S7: Forest plot of "prudent/healthy" dietary pattern and cancer mortality by cancer type 13

SF S8 : Forest plot of "western/unhealthy" dietary pattern and cancer mortality by cancer type........14

Supplementary S8: Sensitivity analysis 15

SF S9: Influence plot of a priori dietary patterns and all-causemortality-all cancer studies 15

SF S10: Influence plot of a priori dietary patterns and breast cancer mortality 16

SF S11: Cumulative meta-analysis of a priori dietary patterns and all-causel mortality 17

SF S12: Cumulative meta-analysis of a priori dietary patterns and breast cancer mortality 18

Supplementary S9: Subgroup analysis by risk of bias 19

SF S13: Forest plot of a priori dietary patterns and all-cause mortality by risk of bias................ 19
SF S14: Funnel plot of a priori dietary patterns and breast cancer mortality by risk of bias......... 20

Supplementary S1: Search strategy

PubMed: ("dietary patterns" OR "a-priori dietary pattern" OR "a posteriori dietary pattern" OR "cluster analysis" OR "factor analysis" OR "diet quality index" OR "Principal Component Analysis" OR (diet* AND index) OR (diet* AND score) OR (nutri* AND index) OR (nutri* AND score) OR "reduced rank regression" OR "Western Pattern" OR" Prudent Pattern" OR "traditional pattern" OR "Mediterranean diet" OR "vegetarian diet" OR "DASH" OR "Nordic diet" OR (diet* AND pattern*) OR (diet* AND habit*) OR (diet* AND quality) OR (food AND quality) OR (nutri* AND quality) OR (food AND pattern*) OR (food AND habit*) OR (nutri* AND habit*) OR (nutri* AND pattern*) OR (eating AND habit*) OR (eating AND pattern*))AND ("Neoplasms"[Mesh] OR cancer) AND (survival OR recurrence OR mortality OR relapse) NOT letter NOT comment NOT editorial AND "humans"[MeSH Terms] AND "english"[Language] NOT letter NOT comment NOT editorial NOT review NOT "meta-analysis" NOT "meta analysis"

Scopus: ((“diet* AND pattern” OR “diet* AND score” OR “nutrition* AND index” OR “diet* AND index OR "diet* habit" OR "eating habit*" OR "Western Pattern" OR "Prudent Pattern" OR "traditional pattern" OR "Mediterranean diet" OR "vegetarian diet" OR "DASH" OR "Nordic diet") AND ("neoplas*" OR "cancer") AND ("survival" OR "recurrence" OR "mortality" OR "rel apse"))

Web of Science: ((diet* AND pattern) OR (diet* AND score) OR (nutrition* AND index) OR (diet* AND index) OR (diet* habit) OR (eating habit*) OR (Western AND Pattern) OR (Prudent AND Pattern) OR (traditional AND pattern) OR (Mediterranean AND diet) OR (vegetarian AND diet) OR (DASH) OR (Nordic AND diet)) AND (neoplas* OR cancer) AND (survival OR recurrence OR mortality OR relapse)

Supplementary S2: Excluded studies

Song M, Wu K, Meyerhardt JA, Yilmaz O, Wang M, Ogino S, Fuchs CS, Giovannucci EL, Chan AT. Low-Carbohydrate Diet Score and Macronutrient Intake in Relation to Survival After Colorectal Cancer Diagnosis. JNCI Cancer Spectr. 2018 Nov;2(4):pky077. doi: 10.1093/jncics/pky077. Epub 2019 Jan 28. PMID: 30734025; PMCID: PMC6350503.

Kunnavuttivanich V, Pramyothin P, Ithimakin S. Association between dietary patterns and disease recurrence in Thai colorectal cancer patients. Medicine (Baltimore). 2020 Mar;99(11):e19522. doi: 10.1097/MD.0000000000019522. PMID: 32176103; PMCID: PMC7440110.

Van Blarigan EL, Fuchs CS, Niedzwiecki D, Zhang S, Saltz LB, Mayer RJ, Mowat RB, Whittom R, Hantel A, Benson A, Atienza D, Messino M, Kindler H, Venook A, Ogino S, Giovannucci EL, Ng K, Meyerhardt JA. Association of Survival With Adherence to the American Cancer Society Nutrition and Physical Activity Guidelines for Cancer Survivors After Colon Cancer Diagnosis: The CALGB 89803/Alliance Trial. JAMA Oncol. 2018 Jun 1;4(6):783-790. doi: 10.1001/jamaoncol.2018.0126. Erratum in: JAMA Oncol. 2019 Apr 1;5(4):579. PMID: 29710284; PMCID: PMC6145685.

Meyerhardt JA, Sato K, Niedzwiecki D, et al. Dietary glycemic load and cancer recurrence and survival in patients with stage III colon cancer: findings from CALGB 89803. J Natl Cancer Inst. 2012; 104:1702–11. [PubMed: 23136358]

Jacobs S, Harmon BE, Ollberding NJ, Wilkens LR, Monroe KR, Kolonel LN, Le Marchand L, Boushey CJ, Maskarinec G. Among 4 Diet Quality Indexes, Only the Alternate Mediterranean Diet Score Is Associated with Better Colorectal Cancer Survival and Only in African American Women in the Multiethnic Cohort. J Nutr. 2016 Sep;146(9):1746-55. doi: 10.3945/jn.116.234237. Epub 2016 Aug 10. PMID: 27511927; PMCID: PMC4997287.

Shen GP, Xu FH, He F, Ruan HL, Cui C, Chen LZ, Zeng YX, Jia WH. Pretreatment lifestyle behaviors as survival predictors for patients with nasopharyngeal carcinoma. PLoS One. 2012;7(5):e36515. doi: 10.1371/journal.pone.0036515. Epub 2012 May 8. PMID: 22590554; PMCID: PMC3348163.

van den Berg MG, Rütten H, Rasmussen-Conrad EL, Knuijt S, Takes RP, van Herpen CM, Wanten GJ, Kaanders JH, Merkx MA. Nutritional status, food intake, and dysphagia in long-term survivors with head and neck cancer treated with chemoradiotherapy: a cross-sectional study. Head Neck. 2014 Jan;36(1):60-5. doi: 10.1002/hed.23265. Epub 2013 Apr 4. PMID: 23559543.

Yang M, Kenfield SA, Van Blarigan EL, Wilson KM, Batista JL, Sesso HD, Ma J, Stampfer MJ, Chavarro JE. Dairy intake after prostate cancer diagnosis in relation to disease-specific and total mortality. Int J Cancer. 2015 Nov 15;137(10):2462-9. doi: 10.1002/ijc.29608. Epub 2015 Jun 3. PMID: 25989745; PMCID: PMC4754664.

Ratjen I, Shivappa N, Schafmayer C, Burmeister G, Nöthlings U, Hampe J, Hébert JR, Lieb W, Schlesinger S. Association between the dietary inflammatory index and all-cause mortality in colorectal cancer long-term survivors. Int J Cancer. 2019 Mar 15;144(6):1292-1301. doi: 10.1002/ijc.31919. Epub 2018 Dec 3. PMID: 30303515.

Arthur AE, Peterson KE, Rozek LS, Taylor JM, Light E, Chepeha DB, Hébert JR, Terrell JE, Wolf GT, Duffy SA; UM Head and Neck SPORE Program. Pretreatment dietary patterns, weight

status, and head and neck squamous cell carcinoma prognosis. Am J Clin Nutr. 2013 Feb;97(2):360-8. doi: 10.3945/ajcn.112.044859. Epub 2012 Dec 26. PMID: 23269814; PMCID: PMC3545683.

Vrieling A, Buck K, Seibold P, Heinz J, Obi N, Flesch-Janys D, Chang-Claude J. Dietary patterns and survival in German postmenopausal breast cancer survivors. Br J Cancer. 2013 Jan 15;108(1):188-92. doi: 10.1038/bjc.2012.521. Epub 2012 Nov 20. PMID: 23169282; PMCID: PMC3553521.

Thomson CA, E Crane T, Wertheim BC, Neuhouser ML, Li W, Snetselaar LG, Basen-Engquist KM, Zhou Y, Irwin ML. Diet quality and survival after ovarian cancer: results from the Women's Health Initiative. J Natl Cancer Inst. 2014 Oct 21;106(11):dju314. doi: 10.1093/jnci/dju314. PMID: 25335480; PMCID: PMC4271032.

Sharma I, Roebothan B, Zhu Y, Woodrow J, Parfrey PS, Mclaughlin JR, Wang PP. Hypothesis and data-driven dietary patterns and colorectal Cancer survival: findings from Newfoundland and Labrador colorectal Cancer cohort. Nutr J. 2018 May 25;17(1):55. doi: 10.1186/s12937-018-0362-x. PMID: 29793493; PMCID: PMC5968482.

Zhu Y, Wu H, Wang PP, Savas S, Woodrow J, Wish T, Jin R, Green R, Woods M, Roebothan B, Buehler S, Dicks E, McLaughlin JR, Campbell PT, Parfrey PS. Dietary patterns and colorectal cancer recurrence and survival: a cohort study. BMJ Open. 2013 Feb 7;3(2):e002270. doi: 10.1136/bmjopen-2012-002270. PMID: 23396503; PMCID: PMC3586110.

Pelser C, Arem H, Pfeiffer RM, Elena JW, Alfano CM, Hollenbeck AR, Park Y. Prediagnostic lifestyle factors and survival after colon and rectal cancer diagnosis in the National Institutes of Health (NIH)-AARP Diet and Health Study. Cancer. 2014 May 15;120(10):1540-7. doi: 10.1002/cncr.28573. Epub 2014 Mar 3. PMID: 24591061; PMCID: PMC4151292.

Carr PR, Jansen L, Walter V, Kloor M, Roth W, Bläker H, Chang-Claude J, Brenner H, Hoffmeister M. Associations of red and processed meat with survival after colorectal cancer and differences according to timing of dietary assessment. Am J Clin Nutr. 2016 Jan;103(1):192-200. doi: 10.3945/ajcn.115.121145. Epub 2015 Nov 25. PMID: 26607936.

Yang B, McCullough ML, Gapstur SM, Jacobs EJ, Bostick RM, Fedirko V, Flanders WD, Campbell PT. Calcium, vitamin D, dairy products, and mortality among colorectal cancer survivors: the Cancer Prevention Study-II Nutrition Cohort. J Clin Oncol. 2014 Aug 1;32(22):2335-43. doi: 10.1200/JCO.2014.55.3024. Epub 2014 Jun 23. PMID: 24958826.

McCullough ML, Gapstur SM, Shah R, Jacobs EJ, Campbell PT. Association between red and processed meat intake and mortality among colorectal cancer survivors. J Clin Oncol. 2013 Aug 1;31(22):2773-82. doi: 10.1200/JCO.2013.49.1126. Epub 2013 Jul 1. PMID: 23816965; PMCID: PMC3718877.

Lang S, Schimansky S, Beynon R, Penfold C, Davies A, Waylen A, Thomas S, Pring M, Pawlita M, Waterboer T, Ness AR. Dietary behaviors and survival in people with head and neck cancer: Results from Head and Neck 5000. Head Neck. 2019 Jul;41(7):2074-2084. doi: 10.1002/hed.25660. Epub 2019 Jan 30. PMID: 30698303; PMCID: PMC7116031.

Sandoval M, Font R, Mañós M, Dicenta M, Quintana MJ, Bosch FX, Castellsagué X. The role of vegetable and fruit consumption and other habits on survival following the diagnosis of oral

cancer: a prospective study in Spain. Int J Oral Maxillofac Surg. 2009 Jan;38(1):31-9. doi: 10.1016/j.ijom.2008.09.004. Epub 2008 Oct 31. PMID: 18951763.

Chan JM, Holick CN, Leitzmann MF, Rimm EB, Willett WC, Stampfer MJ, Giovannucci EL. Diet after diagnosis and the risk of prostate cancer progression, recurrence, and death (United States). Cancer Causes Control. 2006 Mar;17(2):199-208. doi: 10.1007/s10552-005-0413-4. PMID: 16425098.

Dikshit RP, Boffetta P, Bouchardy C, Merletti F, Crosignani P, Cuchi T, Ardanaz E, Brennan P. Lifestyle habits as prognostic factors in survival of laryngeal and hypopharyngeal cancer: a multicentric European study. Int J Cancer. 2005 Dec 20;117(6):992-5. doi: 10.1002/ijc.21244. PMID: 15986425

Supplementary S3: A-priori dietary patterns and all-cause mortality

Figure S1 (SF S1): Galbraith's plot

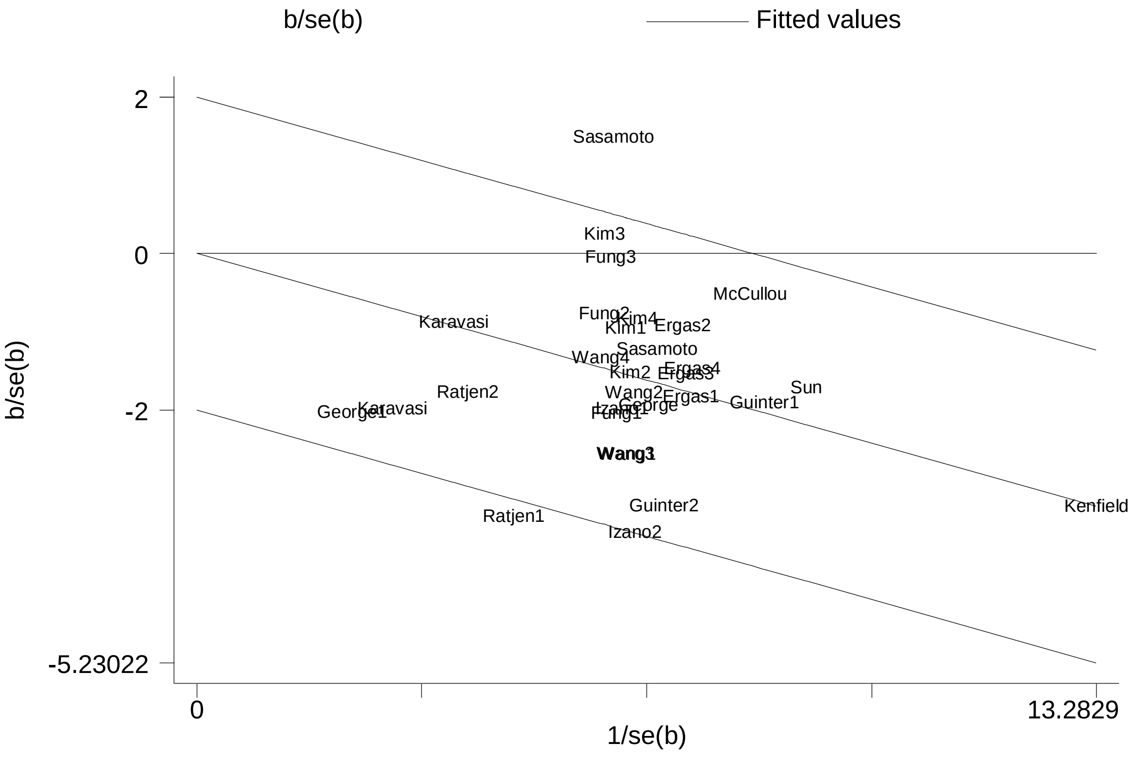

Figure S2 (SF S2): Funnel plot of a priori dietary patterns and all-cause mortality

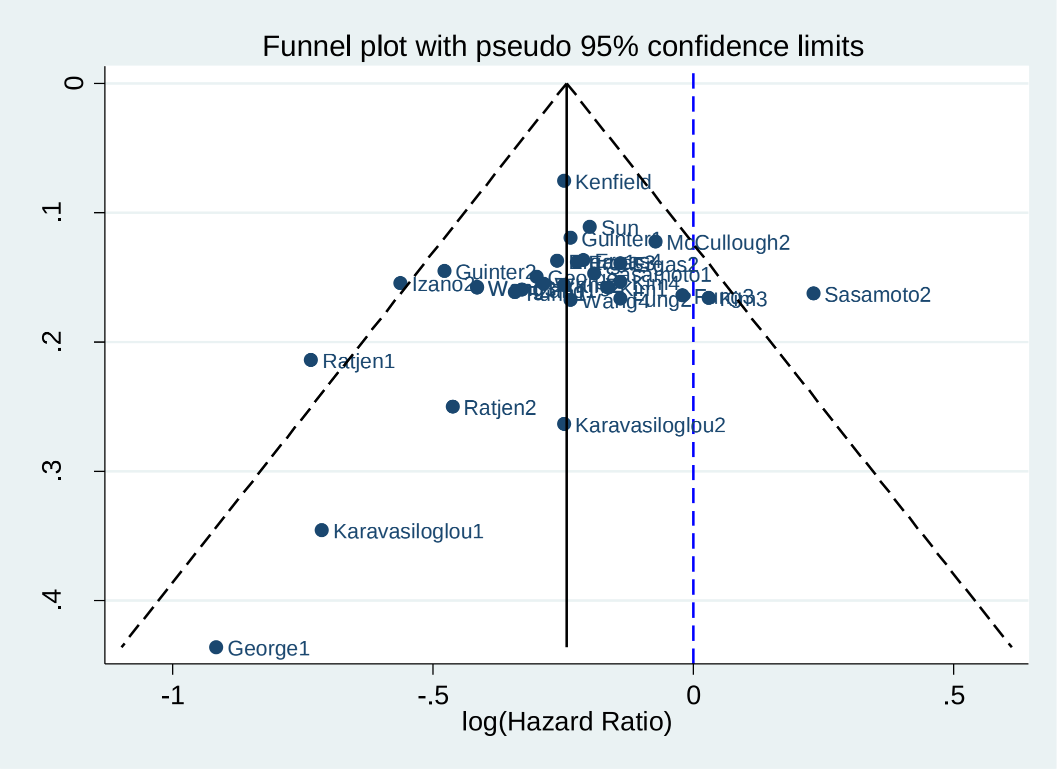

Results from Egger's test (p-value=0.102>0.05) did not indicate any evidence of publication bias

Supplementary S4: A priori dietary patterns and cancer-specific mortality

Figure S3 (SF S3): Forest plot showing the association between highest versus lowest adherence to a priori dietary patterns and cancer-specific mortality by cancer site and overall

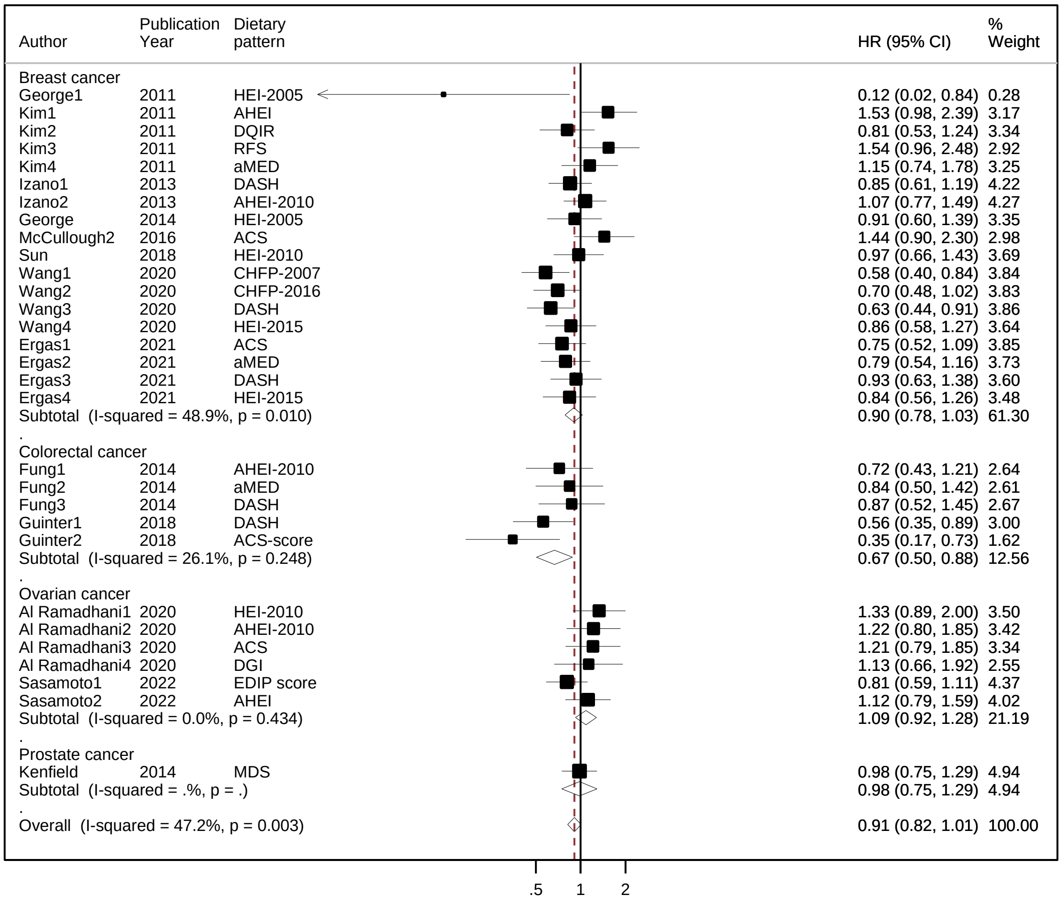

Abbreviations: CHFP-2007: Chinese Food Pagoda-2007, CHFP-2016: Chinese Food Pagoda-2016, MDS: Mediterranean Diet Score, HEI-2015: Healthy Eating Index-2015, HEI-2005: Healthy Eating Index-2005, HEI-2010: Healthy Eating Index-2010, aMED, altMed: alternate Mediterranean Diet Score, MMDS: Modified Mediterranean Diet Score, HNFI: Healthy Nordic Food Index, RFS: Recommended Food Score, DASH: Dietary Approaches to Stop Hypertension, EDIP: Empirical dietary inflammatory pattern, ACS: American Cancer Society, DQIR: Diet Quality Index-Revised, AHEI: Alternate Healthy Eating Index, AHEI-2010: Alternate Healthy Eating Index-2010, DGI: Australian Dietary Guideline Index

Figure S4 (SF S4): Funnel plot of a priori dietary patterns and cancer-specific mortality among all studies

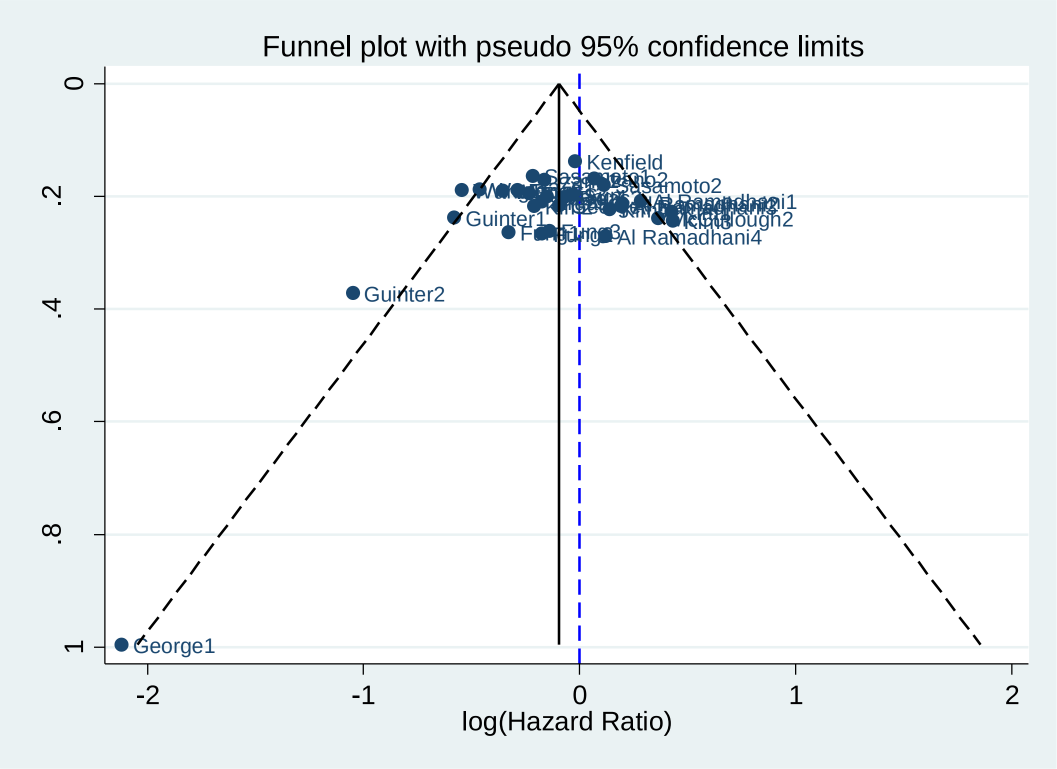

Results from Egger's test (p-value=0.281>0.05) indicate no evidence of publication bias.

Supplementary S5: A priori dietary patterns and cancer-specific mortality - Further analysis of breast cancer studies

Figure S5 (SF S5): Funnel plot of a priori dietary patterns and breast cancer mortality

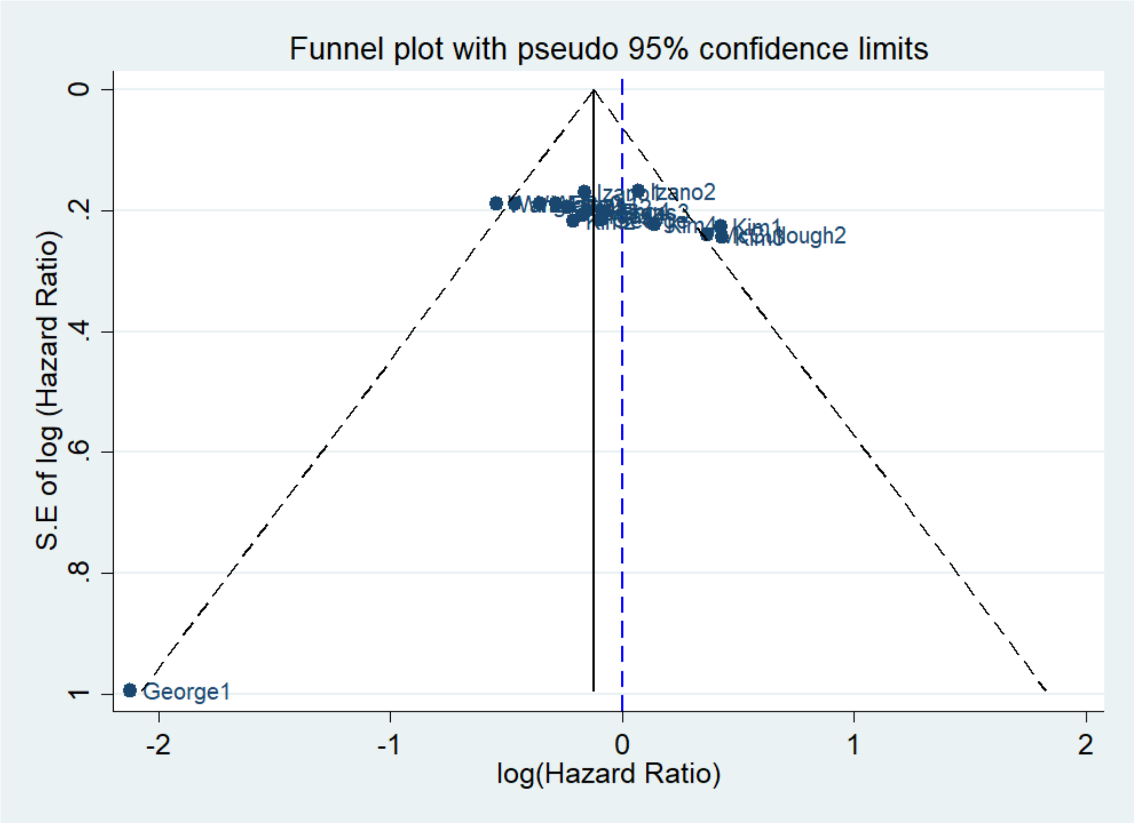

Results from Egger's test (p-value=0.838>0.05) indicate no evidence of publication bias.

Supplementary S6: A priori dietary patterns and cancer-specific mortality - Further analysis of colorectal cancer studies

Figure S6 (SF S6): Funnel plot of a priori dietary patterns and colorectal cancer mortality

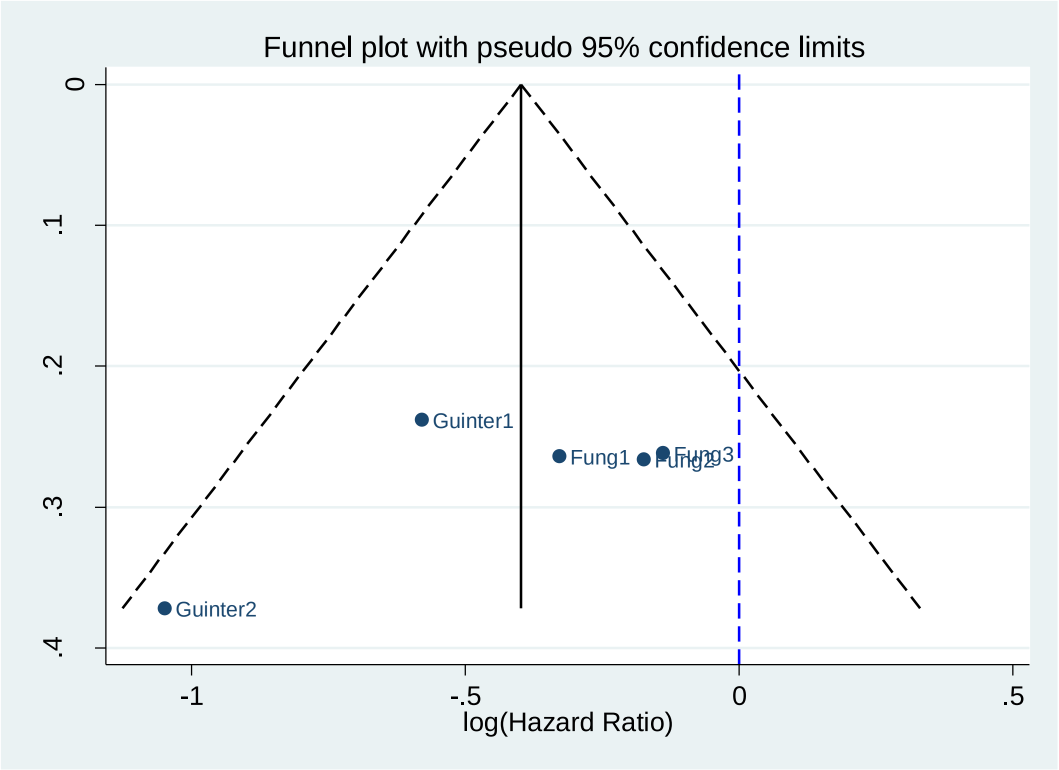

Due to the small number of studies (n<10), Egger's test, Galbraith's plot, Influence plot, and Cumulative meta-analysis were not performed.

Supplementary S7: A posteriori dietary patterns and cancer-specific mortality

Figure S7 (SF S7): Forest plot showing the association between highest versus lowest adherence to a “prudent/healthy” dietary pattern and cancer-specific mortality by cancer site and overall, among cancer survivors

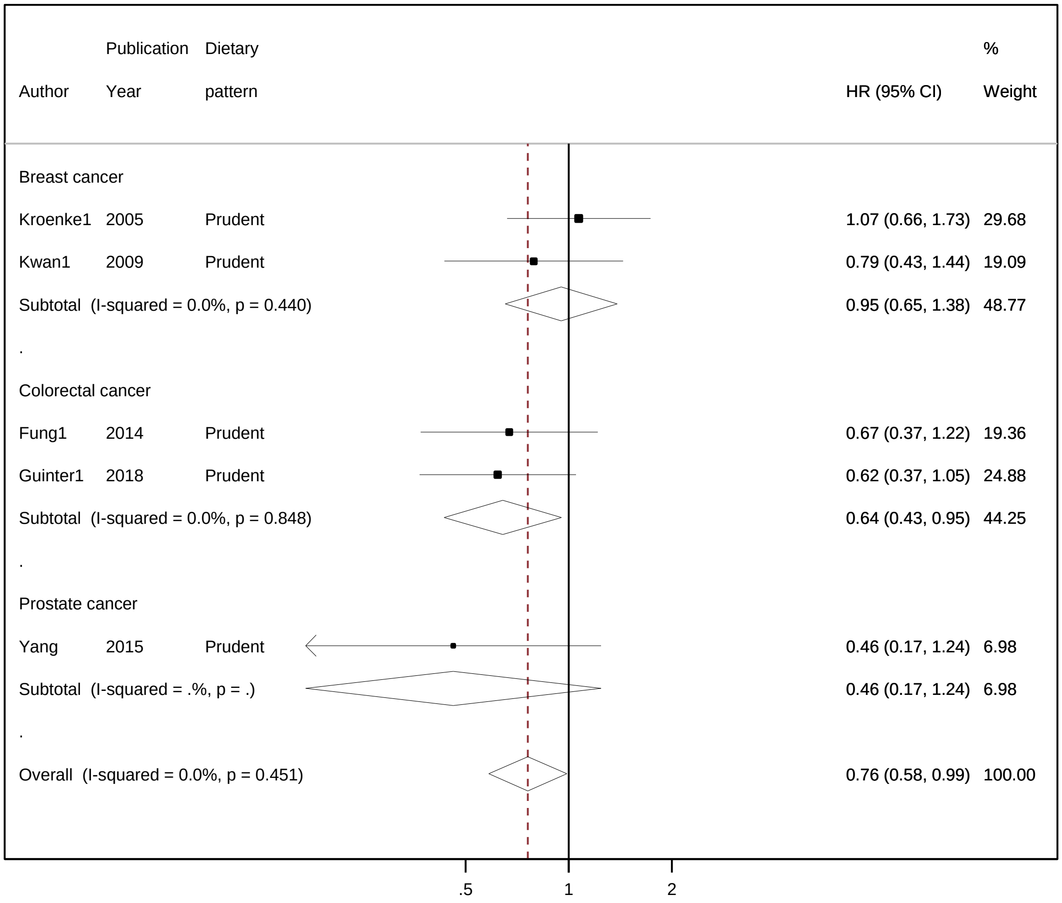

Figure S8 (SF S8): Forest plot showing the association between highest versus lowest adherence to "western/unhealthy" dietary pattern and cancer-specific mortality by cancer type and overall, among cancer survivors

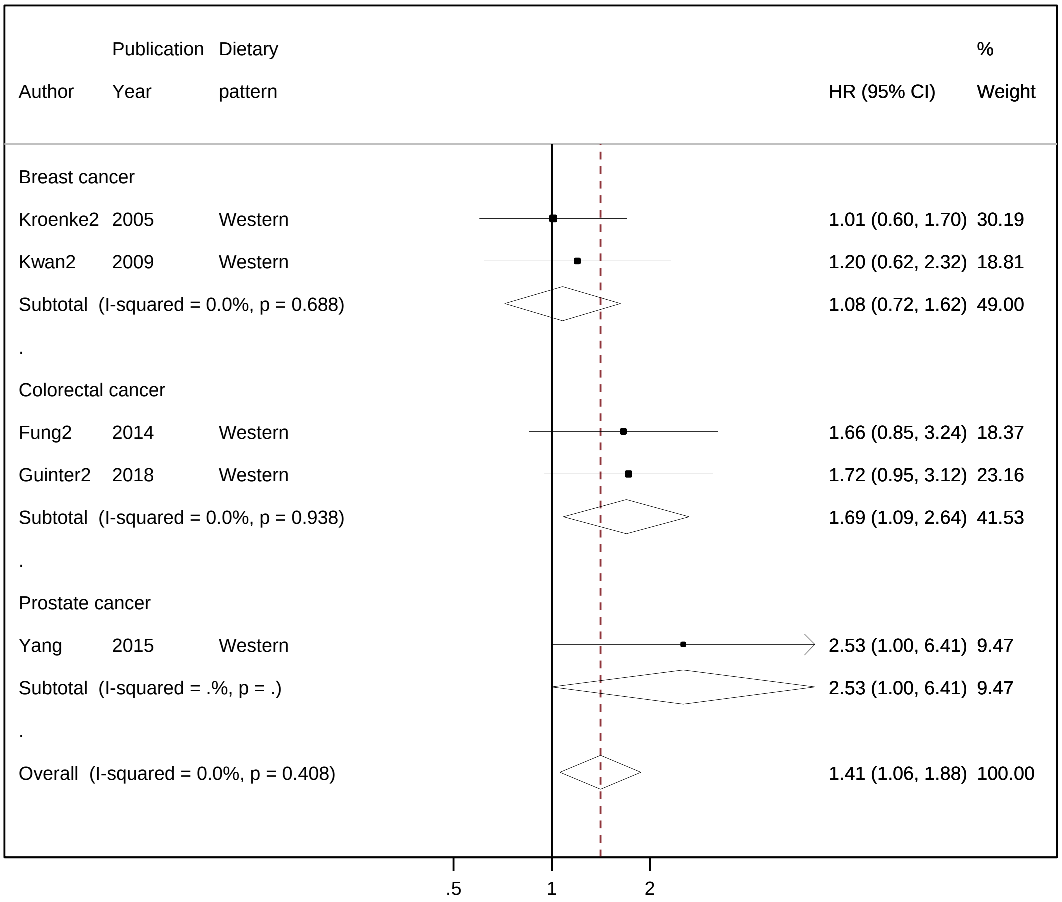

Supplementary S8: Sensitivity analysis

Figure S9 (SF S9): Influence plot of a priori dietary patterns and all-cause mortality-all studies

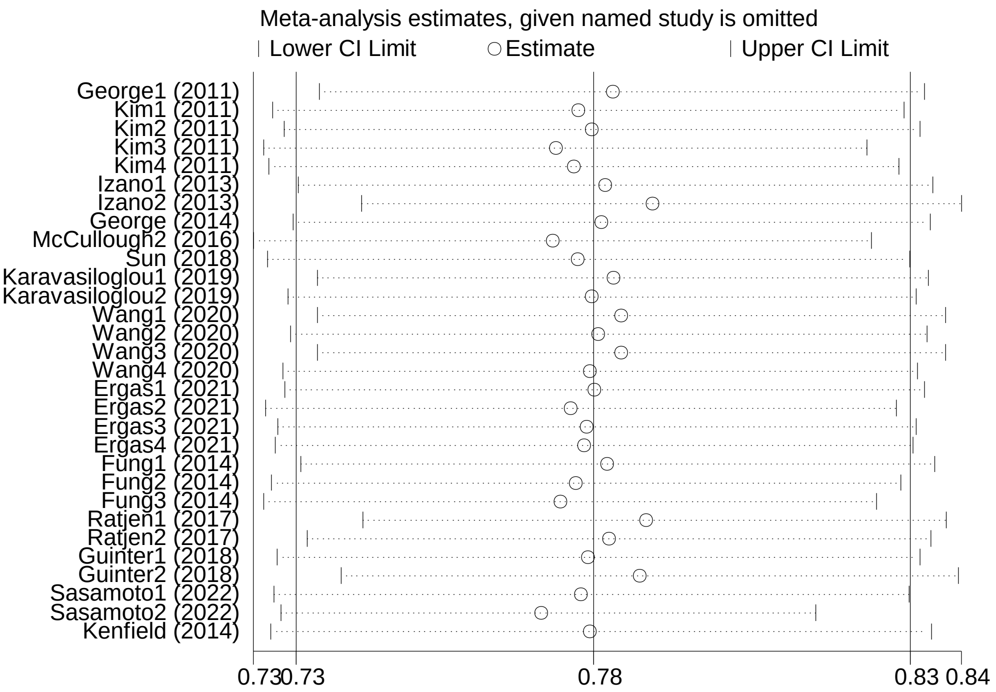

Figure S10 (SF S10): Influence plot of a priori dietary patterns and breast cancer mortality

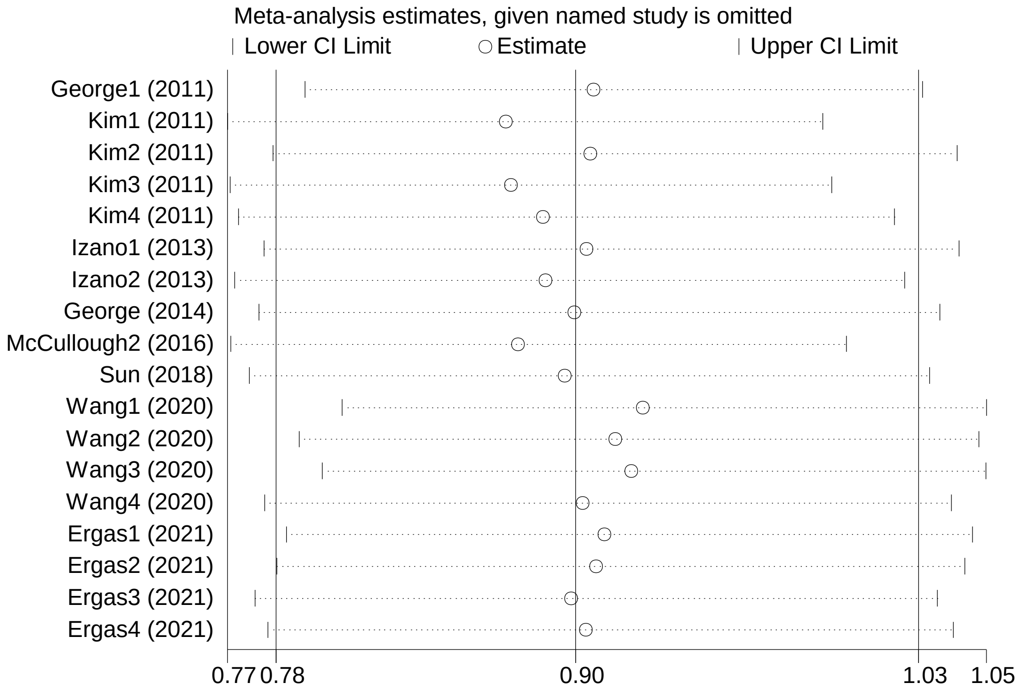

Figure S11 (SF S11): Cumulative meta-analysis of a priori dietary patterns and all-cause mortality

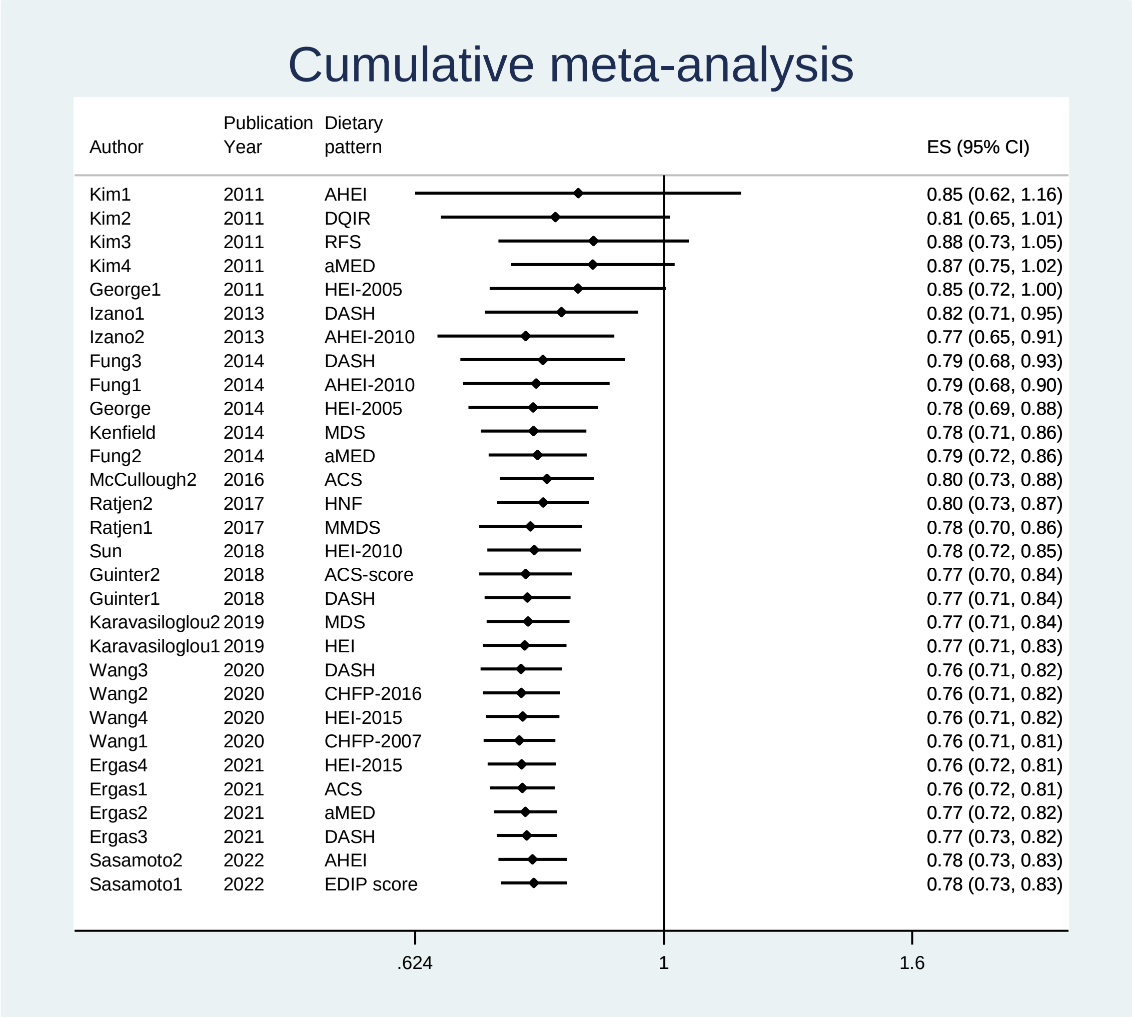

Figure S12 (SF S12): Cumulative meta-analysis a priori dietary patterns and breast cancer mortality

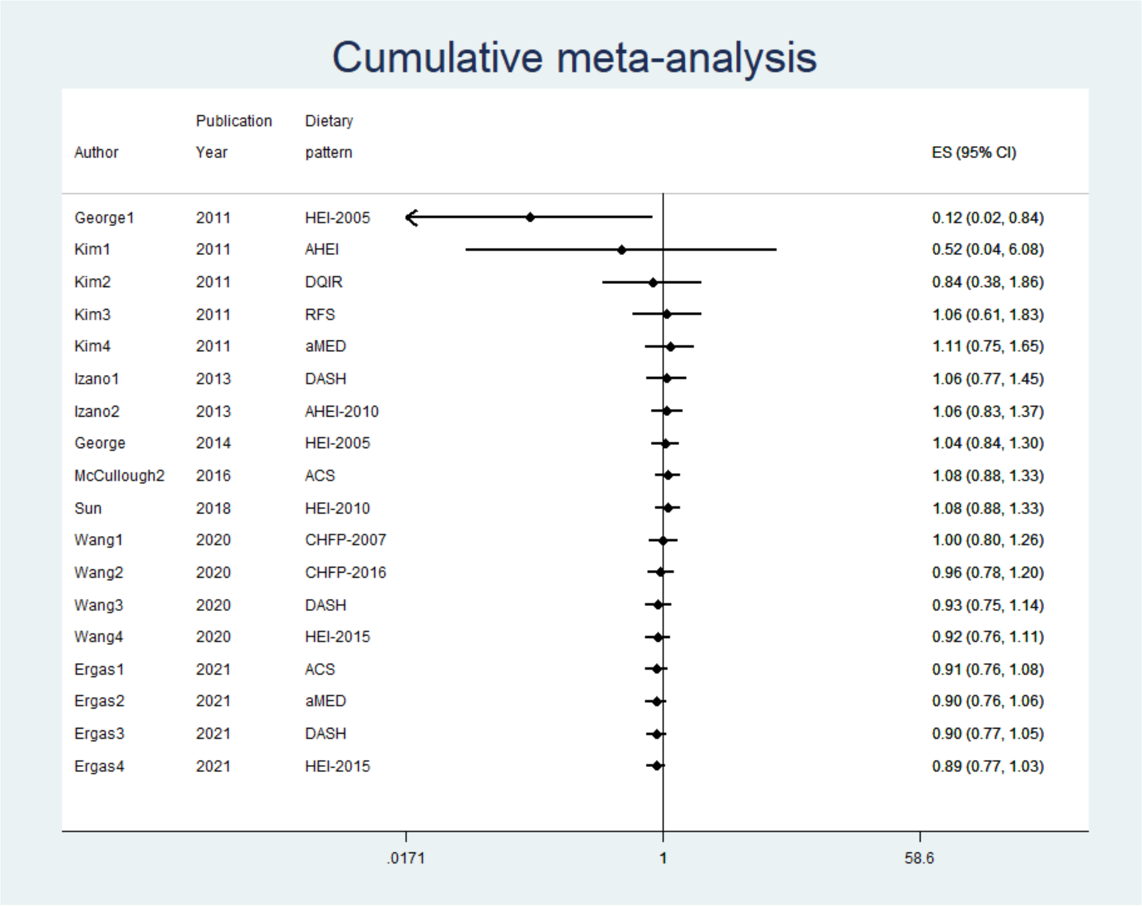

Supplementary S9: Subgroup analysis by risk of bias

Figure S13 (SF S13): Forest plot showing the association between highest vs lowest adherence to a priori dietary patterns and all-cause mortality among cancer survivors by risk of bias

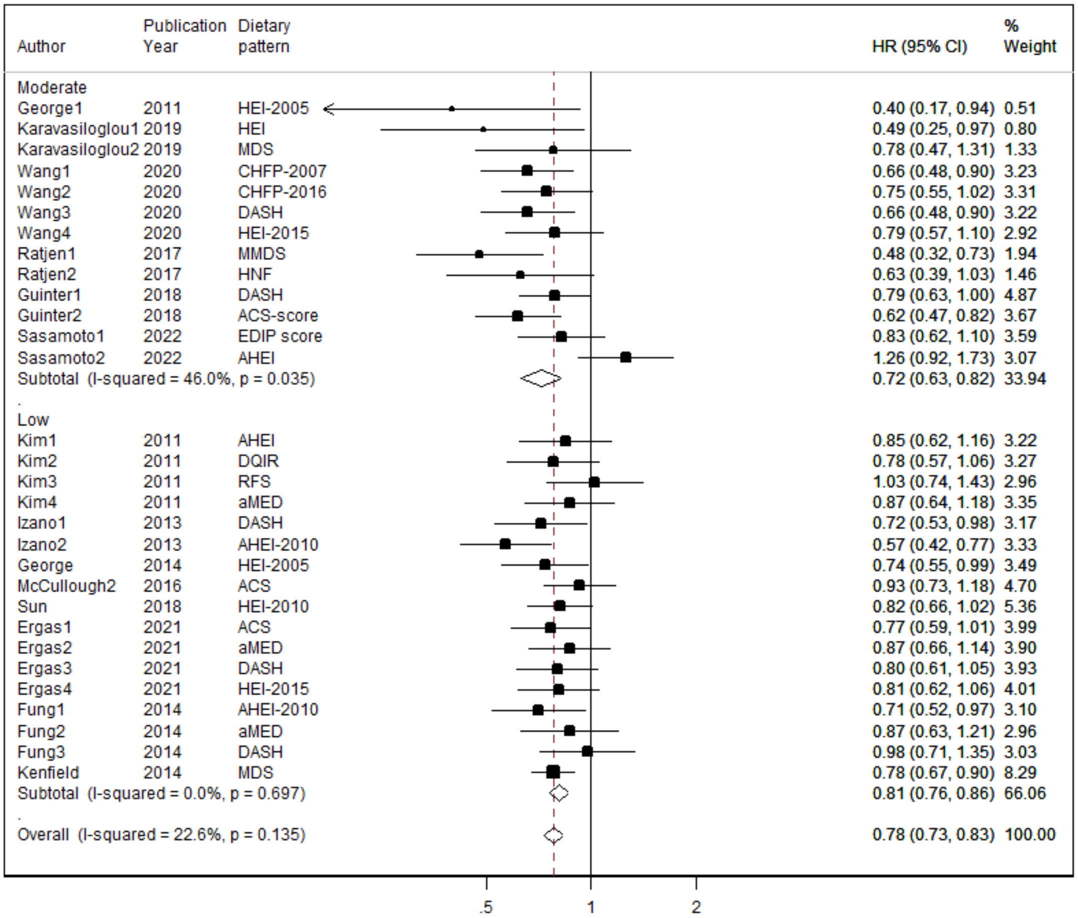

Figure S14 (SF S14): Funnel plot of a-priori dietary patterns and all-cause mortality by risk of bias

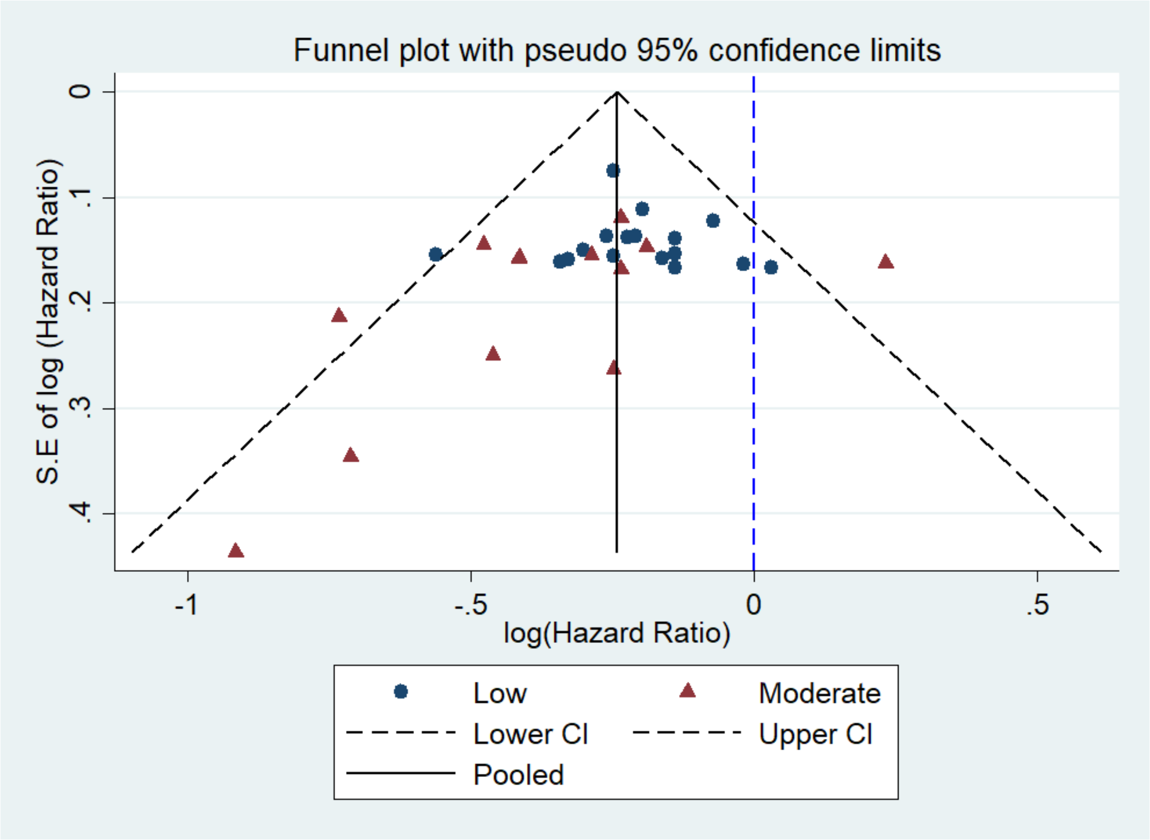

Supplement: Supplementary file 1 [file nutrients-15-03860-s001.zip › nutrients-2581982-supplementary.pdf]
